# Stimuli-Responsive Polypeptide Nanoparticles for Enhanced DNA Delivery

**DOI:** 10.3390/molecules27238495

**Published:** 2022-12-02

**Authors:** Olga Korovkina, Dmitry Polyakov, Viktor Korzhikov-Vlakh, Evgenia Korzhikova-Vlakh

**Affiliations:** 1 Institute of Chemistry, Saint-Petersburg State University, Universitetsky pr. 26, 198504 St. Petersburg, Russia; 2 Institute of Experimental Medicine, Acad. Pavlov Street 12, 197376 St. Petersburg, Russia; 3 Institute of Macromolecular Compounds, Russian Academy of Sciences, Bolshoy pr. 31, 199004 St. Petersburg, Russia

**Keywords:** pH and redox-responsive delivery systems, cross-linked nanoparticles, polypeptides, gene delivery

## Abstract

The development of non-viral delivery systems for effective gene therapy is one of the current challenges in modern biomedicinal chemistry. In this paper, the synthesis of pH- and redox-responsive amphiphilic polypeptides for intracellular DNA delivery is reported and discussed. Two series of polypeptides consisting of L-lysine, L-phenylalanine, L-histidine, and L-cysteine as well as the same amino acids with L-glutamic acid were synthesized by a combination of copolymerization of N-carboxyanhydrides of α-amino acids and post-polymerization modification of the resulting copolymers. The presence of histidine provided pH-sensitive properties under weakly acidic conditions specific to endosomal pH. In turn, the presence of cysteine allowed for the formation of redox-responsive disulfide bonds, which stabilized the self-assembled nanoparticles in the extracellular environment but could degrade inside the cell. The formation of intraparticle disulfide bonds resulted in their compactization from 200–250 to 55–100 nm. Empty and pDNA-loaded cross-linked nanoparticles showed enhanced stability in various media compared to non-crosslinked nanoparticles. At the same time, the addition of glutathione promoted particle degradation and nucleic acid release. The delivery systems were able to retain their size and surface charge at polypeptide/pDNA ratios of 10 or higher. GFP expression in HEK 293 was induced by the delivery of pEGFP-N3 with the developed polypeptide nanoparticles. The maximal transfection efficacy (70%) was observed when the polypeptide/pDNA ratio was 100.

## 1. Introduction

Gene therapy is one of the powerful tools used to treat many disorders, including cancer, genetic and infectious diseases. The main mechanisms conceivable in gene therapy are based on replacing the damaged gene with a healthy copy or inactivating the damaged gene [1,2,3]. Such therapy provides an opportunity to treat a wide spectrum of diseases at the stage of transcription and translation. The successful treatment is based on transferring one or more therapeutic nucleic acids into the patient’s cells. However, nucleic acids are negatively charged and cannot enter successfully into the cells. Moreover, they are not stable in the free state and are easily attacked by nucleases in vivo. This problem can be overcome by using gene delivery systems.

The development of safe non-viral systems for controlled intracellular drug delivery is an important task of modern science. Currently, nucleic acid delivery systems are represented by lipid nanoparticles [4,5,6], lipoplexes that are cationic liposomes [7], cationic polymers (poly(ethyleneimine), poly(amidoamine), polypeptides etc.) that form polyplexes with DNA/RNA [8,9,10,11,12,13], and micelles [14,15,16].

Among other classes of polymer delivery systems, L-polypeptides are a promising group of biodegradable polymers that have found many applications in biomedicine. Cationic poly(amino acids) such as poly(lysine), poly(ornithine) and poly(arginine) are the first polypeptides considered as non-viral candidates for DNA delivery [17,18]. However, the toxicity of cationic poly(amino acids), as well as the limited release of nucleic acids from polyplexes with these polypeptides, encourage their combination with non-toxic and stimuli-responsive moieties.

Stimuli-responsive carriers are a class of next-generation delivery systems [19,20]. The use of polymers, which can respond to various physiological or pathological stimuli (pH, temperature, glutathione concentration, enzymes, etc.), is a promising approach to controlled gene delivery [21]. For instance, to make the system pH-sensitive, histidine (His) can be introduced into polypeptide structure. The secondary amine of the imidazole ring is protonated in acidic conditions (pKa~6). This property of His provides the exit of the delivery system from the endosome to the cytoplasm due to the so-called “proton sponge effect” [22]. It is based on the protonation of the secondary amino groups in the acidic environment of the endosome (pH 5–6) after capture of the delivery systems by cells. This protonation prevents normal endosome acidification and, finally, its transformation into a lysosome, due to the pumping of more protons and an increased influx of Cl^−^ and water. This buffering leads to destruction of the late endosomes and release of the content into the cytoplasm due to both osmotic swelling and nanoparticle swelling caused by increased polymer chain repulsion. Some histidine and lysine copolymers have been reported as promising pH-sensitive carriers for nucleic acids [23,24,25]. In this case, the primary amino groups in lysine ensure efficient binding to nucleic acids and better penetration through the cell membrane, while histidine provides endosomal buffering and escape from the endosome into the cytoplasm [26,27]. Recently, the positive effects of negatively charged units of glutamic acid [28] and histidine in the cationic polypeptide to develop pH-sensitive polymers for efficient siRNA delivery have been demonstrated [29].

Furthermore, non-toxic complexes polymer/DNA of certain size and surface charge [30] must be stable in the extracellular space and able to degrade inside the cell to be successful for the application in vivo. One of the approaches to prolong the stability of such complexes is the formation of intraparticle cross-links. Intraparticle cross-linking should obstruct the release of DNA from the complex with polymer in the presence of competing polyanions (proteins, heparin, etc.) in the extracellular environment. At the same time, disruption of cross-links inside a cell must ensure the efficient release of nucleic acid from the polymeric delivery system via displacement by competing polyanions. The most important natural antioxidant that regulates many processes as a reducing agent is glutathione (GSH) [31]. One of the main biological functions of GSH is the reduction of disulfide bonds in proteins to thiols. It is known that cytosol GSH concentration (2–10 mM) is about three orders of magnitude higher than in blood plasma (2–20 µM) [32]. Such a difference in reducing agent concentrations serves as a convenient differentiator between the extracellular and intracellular space, as well as between healthy and tumor tissues. In particular, in vivo studies showed that GSH concentration in cancer tissues is four times higher than in healthy mice tissues [33]. Thus, the redox-responsive properties of disulfide bonds in the physiological environment make them very attractive to prepare the delivery systems for intracellular delivery.

Recently, Ren at al. described the synthesis of stabilized polyplexes containing poly(lysine) and several cysteine residues [30]. After oxidation of thiol groups, intermolecular glutathione-sensitive disulfide cross-links were formed. The cross-linked complexes demonstrated significantly higher transfection efficiency compared to their non-cross-linked counterparts. Matsumoto et al. [34] reported the synthesis of thiolated block-copolymer of PEG with poly(lysine). The cross-linked micelles were stable in buffer solutions and at high salt concentrations (~0.5 M NaCl), but quickly broke down in the presence of glutathione. A series of other thiolated micelles with a cross-linked core demonstrated that the balanced ratio of cationic charge density to disulfide cross-linking plays a crucial role in nucleic acid release and overall transfection efficiency [35].

**Figure 1:**
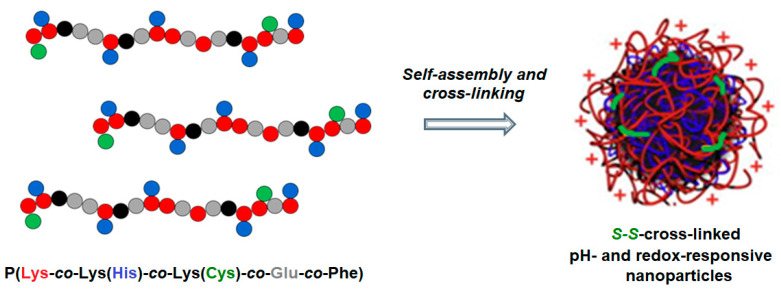
pH- and redox-responsive nanoparticles designed for delivery of nucleic acids.

Here, a preparation of novel redox- and pH-sensitive polypeptide nanoparticles (Figure 1) for DNA delivery that offer the advantage of intraparticle cross-linking is developed and discussed. The synthesis of amphiphilic pH- and redox-responsive polypeptides, representing copolymers of L-lysine, L-glutamic acid, L-phenylalanine, L-histidine and L-cysteine, was carried out by combination of ring-opening copolymerization of N-carboxyanhydrides of α-amino acids and post-polymerization modification of the polypeptides obtained. The polypeptide structure based on L-isomers makes the developed carriers sensitive to enzymes providing biodegradability of the delivery systems. The presence of glutamic acid and histidine in polypeptides provides pH-sensitivity, while the presence of cysteine allows for the formation of redox-responsive disulfide bonds. Being amphiphiles, copolymers are able to self-assembly into nanoparticles. The stabilization of nanoparticles via S-S cross-linking was carried out after the formation of complexes between the copolymer and DNA. Such stabilized complexes showed good stability in different media, such as buffer solution and cell culture media with serum.

The cytotoxicity of nanoparticles was assessed using the MTT assay, and the applicability as pDNA delivery systems was evaluated by the induction of GFP expression in HEK 293.

## 2. Results and Discussion

### 2.1. Synthesis and Characterization of Polypeptides

During our recent studies, the hypothesis on the positive effect of the negatively charged amino acid (i.e., glutamic acid) in copolymers containing lysine was proven [28,29]. It was shown that the presence of the glutamic acid in lysine-enriched copolymer reduces electrostatic interactions between lysine and siRNA and promotes better release of nucleic acid. The aim of the work was to compare the properties of previously developed pH-sensitive polypeptides and novel pH- and redox-responsive systems for pDNA delivery. The synthesis of the stimuli-responsive polypeptides included several steps: (1) synthesis of parent polypeptides consisting of (a) L-lysine (Lys, K), L-glutamic acid (Glu, E) and L-phenylalanine (Phe, F), and of (b) L-lysine and L-phenylalanine via ring-opening copolymerization of N-carboxyanhydrides of corresponding α-amino acids; (2) modification of parent polypeptides with L-histidine (His, H); and (3) modification of histidine-containing polypeptides with L-cysteine (Cys, C).

**Figure 2:**
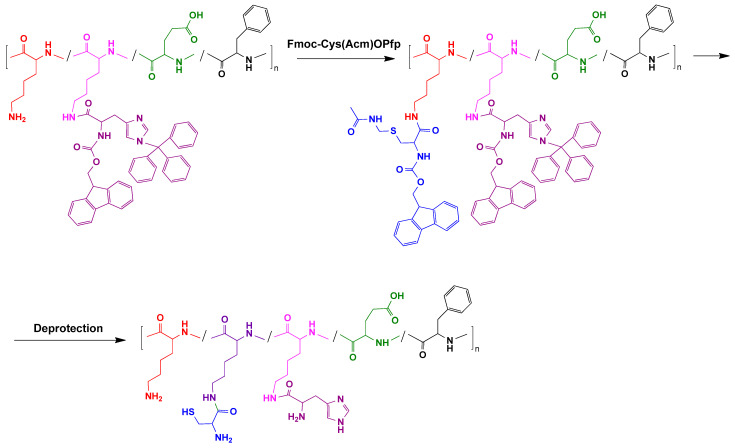
Scheme of the synthesis of a cysteine-containing polypeptide by the example of the P(Lys-*co*-Lys(His)-*co*-Glu-*co*-Phe) modification.

Amino groups of lysine were utilized for post-polymerization modification of parent polypeptide. As an example, Figure 2 illustrates the scheme of polypeptide modification with cysteine residues. At this step, the amino- and imidazole groups of histidine were protected in order to perform selective covalent binding of protected cysteine pre-activated carboxyl groups and amino groups of lysine. The amount of cysteine was set as 7 and 12 mol% from the total lysine content in the copolymer. After synthesis, all protective groups of histidine and cysteine were removed to obtain amphiphilic pH- and redox-sensitive copolymers. Using this strategy, two copolymers were synthesized and characterized: P(Lys-*co*-Lys(His)-*co*-Lys(Cys)-*co*-Glu-*co*-Phe) (further abbreviated as K(HC)EF) and P(Lys-*co*-Lys(His)-*co*-Lys(Cys)-*co*-Phe) (further abbreviated as K(HC)F).

According to static light scattering, the weight average molecular weight (*M_w_*) for the parent copolymers, namely P(Lys-*co*-Glu-*co*-Phe) (KEF) and P(Lys-*co*-Phe) (KF), were 14,600 (R_*h-D*_ = 1.3 nm) and 10,100 (R_*h-D*_ = 1.0 nm). The composition of parent copolymers was determined by quantitative HPLC analysis of amino acids, obtained after total hydrolysis of polypeptides. The rate of lysine modification with histidine was calculated from ^1^H NMR spectra of deprotected copolymers. The efficacy of polymer modification with histidine was about 85%. The empirical content of the thiol groups in a freshly deblocked copolymer was established using Ellman’s test. The efficacy of covalent modification with cysteine was in the range of 65–75%. The composition of the final polypeptides is presented in Table 1.

### 2.2. Preparation and Characterization of Cross-Linked Nanoparticles

Being amphiphilic, the polypeptides obtained demonstrate a tendency to self-assemble in aqueous medium. The preparation of nanoparticles’ dispersion included (1) the gradient solvent inversion (dialysis) from organic solvent to water, (2) freeze-drying and (3) further redispersion of the weighted sample in water or buffer solution under short-term ultrasonic exposure (15 s). During the first step, the self-assembly was stimulated by the low solubility of the hydrophobic part of the polypeptide in water and, consequently, the increased hydrophobic interactions in organic water and water. Eventually, the polypeptides are self-assembled into nanoparticles to minimize the contact of phenylalanine with water and to maximize the exposure of hydrophilic amino acids to the surface of the particle. At the second step, this structure is fixed by lyophilization and is suitable for storage (at 4 °C) and weighing the amount of interest to prepare the required dispersion at the third step.

The hydrodynamic diameter (*D_H_*) for the S-S-cross-linked polymer nanoparticles was investigated by dynamic light scattering (DLS). Zeta-potential was determined by electrophoretic light scattering (ELS, or otherwise laser Doppler electrophoresis). The nanoparticles formed from the parent polypeptides have hydrodynamic diameters of 290 nm (PDI = 0.33) and 210 nm (PDI = 0.24) for P(Lys-*co*-Glu-*co*-Phe) and P(Lys-*co*-Phe), respectively. Modification with histidine favored the decrease in *D_H_* to 180 nm (PDI = 0.21) and 150 nm (PDI = 0.22) for P(Lys-*co*-Lys(His)-*co*-Glu-co-Phe) and P(Lys-*co*-Lys(His)-*co*-Phe), respectively. This decrease in *D_H_* is explained by additional hydrophilization of the polypeptides and improvement in their solubility in aqueous media. In turn, the introduction of cysteine and cross-linking of the nanoparticles resulted in the formation of compact nanoparticles with an average hydrodynamic diameter in the range of 55–100 nm (Table 2).

**Figure 3:**
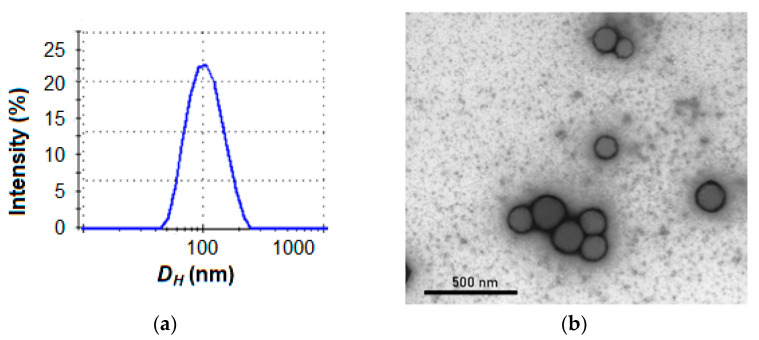
DLS analysis (**a**) and TEM microphotograph (**b**) of sample K(HC)F-2.

At the same time, PDI for Cys-containing nanoparticles varied from 0.37 to 0.43. According to DLS, the obtained nanoparticles had a monomodal particle size distribution (Figure 3a). TEM analysis of the same sample proved the formation of spherical particles 80–130 nm in size, which correlate with DLS data (Figure 3b).

In all cases, the nanoparticles had a high positive charge (Table 2), which along with their small size, indicates their good colloidal stability.

### 2.3. Stability of Nanoparticles in Presence of Proteins and Glutathione

Biological fluids containing negatively charged proteins (e.g., blood plasma) can disorder nanoparticles and replace negatively charged cargo due to polyelectrolyte interactions. Given this, the stability of the particles in the complex media is one of the key properties for the gene delivery systems.

**Figure 4:**
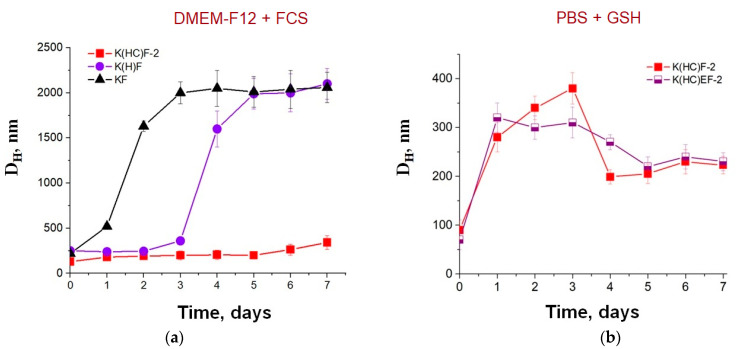
Stability of different nanoparticles incubated at 37 °C (monitoring by DLS) in cell culture medium containing proteins (DMEM-F12 + FCS) (**a**) and cross-linked nanoparticles in the 0.01 M phosphate buffer saline (PBS) solution containing glutathione (GSH, 10 mM) (**b**).

As a complex medium to evaluate the stability of the developed nanoparticles, the DMEM-F12 cell culture medium containing fetal calf serum (FCS) was utilized. For comparison, polypeptides of three series were examined: parent KF, its histidine-containing derivative (K(H)F), and both histidine- and cysteine-containing derivative (K(HC)F). The latter was stabilized with disulfide bonds. All samples were incubated at 37 °C for 7 days. The stability of nanoparticles during the experiment was monitored by DLS. It was found that the uncross-linked KF- and K(H)F-based nanoparticles began to aggregate after 1 or 3 days, respectively (Figure 4a). The histidine-containing sample (K(H)F) kept its original hydrodynamic diameter longer than the parent polypeptides (KF). At the same time, cross-linked nanoparticles retained their size in the protein-containing medium during the experiment due to additional stabilization with disulfide bonds.

In order to assess the redox sensitivity of the S-S-stabilized polypeptide nanoparticles, their stability in a 10 mM glutathione (GSH) solution in PBS simulating the intracellular environment was also examined. As expected, in the presence of GSH, an increase in the hydrodynamic diameter of nanoparticles with disulfide bridges was observed already after 1 day of incubation, indicating that the S-S bonds were cleaved (Figure 4b). On the following days, the hydrodynamic diameter decreased to values of about 200–250 nm due to rearrangement of copolymers. The size of nanoparticles after disulfide bond breaking was similar to the size of uncross-linked parent self-assembled nanoparticles (see Section 2.2).

### 2.4. Characterization of pDNA-Loaded Cross-Linked Polypeptide Nanoparticles

Preparation of the pDNA delivery formulation included several steps: (1) dispersion of polypeptide nanoparticles in water or buffer solution; (2) reduction of S-S bonds that may form in polypeptides as a result of thiol oxidation during nanoparticle storage; (3) complexation of negatively charged pDNA with positively charged polypeptide nanoparticles through electrostatic interactions between phosphate groups and free lysine or histidine amino groups; (4) purification and stabilization of loaded nanoparticles; and (5) thiol oxidation to crosslink nanoparticles.

**Figure 5:**
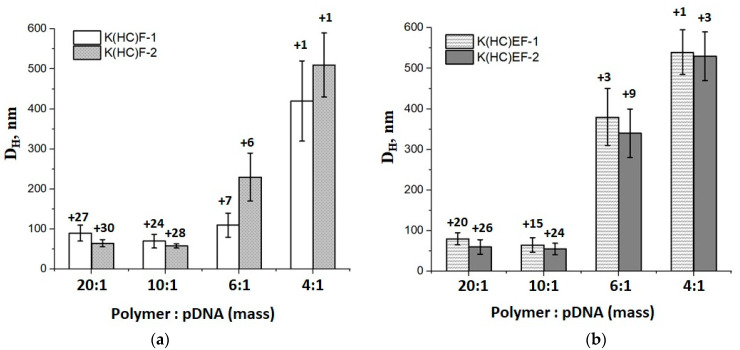
Hydrodynamic diameters and ζ-potential values (above the bars on histograms) for cross-linked pDNA delivery systems obtained at various polypeptide:pDNA mass ratios for two kinds of polypeptides with different cysteine content: (**a**) K(HC)F-1 and K(HC)F-2; (**b**) K(HC)EF-1 and K(HC)EF-2.

A series of solutions with different mass ratios of polypeptide:pDNA and stabilized by S-S bridges were prepared to analyze the hydrodynamic diameter and surface ζ-potential of the complexes. Figure 5 shows the results obtained for two series of copolymers: K(HC)EF (a) and K(HC)F (b); the values of ζ-potential are on the top of each bar in the histograms. As seen, the compact (up to 100 nm) cross-linked complexes with sufficient-for-stabilization surface charge (in the range of 15–30 mV) were prepared at ratios polypeptide:pDNA = 20:1 and 10:1 for both polypeptide series. Moreover, a proper hydrodynamic diameter (up to 250 nm) was also observed for the K(HC)F copolymer, which does not contain negatively charged glutamic acid at the polypeptide:pDNA ratio equal to 6:1. In turn, an increase in the amount of pDNA up to the ratio of polypeptide:pDNA = 4:1 was accompanied by the considerable increase in the hydrodynamic diameter and loss of surface charge due to compensation of the negative and positive charges.

**Figure 6:**
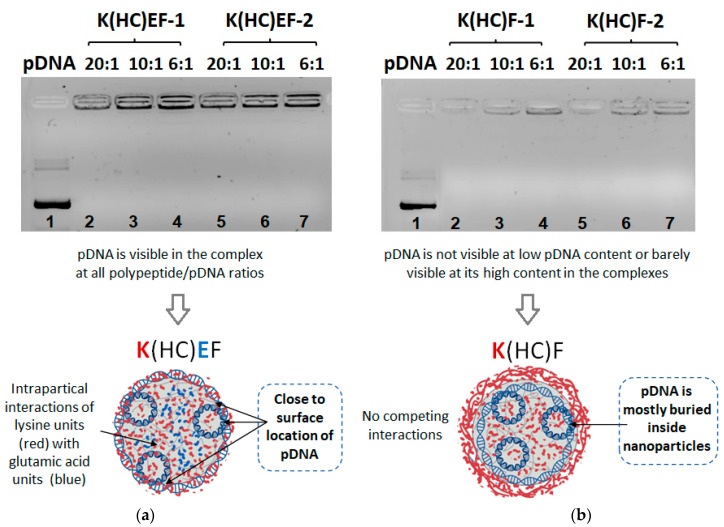
Efficiency and features of pDNA loading into K(HC)EF (**a**) and K(HC)F (**b**) polypeptide nanoparticles followed by S-S cross-linking.

Additionally, agarose gel electrophoresis was used to evaluate the binding efficiency of pDNA to polypeptides forming nanoparticles. The most successful ratios established by DLS, namely polypeptide:pDNA = 20:1, 10:1 and 6:1, were selected for the analysis by gel electrophoresis. As seen from the gel images shown in Figure 6, both kinds of cross-linked nanoparticles, distinguished by the presence (K(HC)EF series, Figure 6a) or absence (K(HC)F series, Figure 6b) of glutamic acid, exhibited strong binding of pDNA to polypeptides. However, a comparison of the zones for K(HC)EF- and K(HC)F-based cross-linked nanoparticles revealed a difference between pDNA loading into different nanoparticles. In particular, pDNA loaded at polypeptide:pDNA ratio = 20:1 in K(HC)F (polypeptides without glutamic acid) was not visualized in the gel (Figure 6b, lanes 2 and 5), which indicates the burial of nucleic acid inside nanoparticles. When pDNA loading was increased (polypeptide:pDNA ratios of 10:1 and 6:1), a small band corresponding to bound pDNA could be detected (Figure 6b, lanes 3,4 and 6,7). Thus, with more loaded pDNA, the nucleic acid is localized both inside the nanoparticles and near the nanoparticle surface. In contrast, for K(HC)EF (polypeptides containing glutamic acid), the surface-bound pDNA is detected at all polypeptide:pDNA ratios, and the band intensity increased with the growth of pDNA amount (Figure 6a). Such a difference can be explained by the involvement of glutamic acid, presenting in the polypeptide, in an additional electrostatic interaction with the lysine units within the nanoparticles, which probably reduces the ability to load the nucleic acid inside.

### 2.5. Stability of pDNA Delivery Systems in the Presence of Competing Polyanions and Reducing Agent

**Figure 7:**
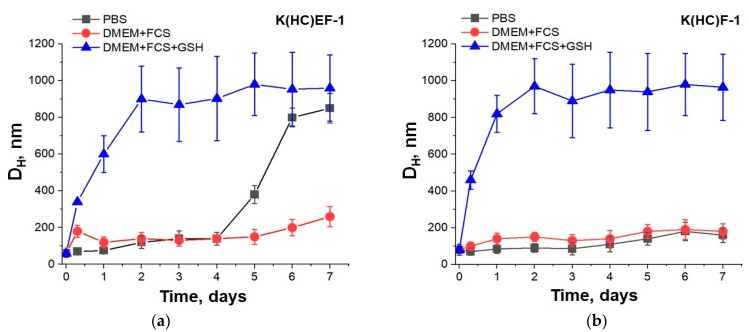
Stability of pDNA-loaded cross-linked nanoparticles based on K(HC)EF (**a**) and K(HC)F (**b**) polypeptides (incubation at 37 °C, DLS monitoring; polypeptide:pDNA ratio was equal to 20:1).

The stability of pDNA-loaded cross-linked nanoparticles was evaluated by DLS for 7 days in different media, namely in buffer solution (0.01 M PBS), cell culture medium containing proteins (DMEM + FCS) and cell culture medium containing proteins and glutathione (DMEM + FCS + GSH) (Figure 7). As expected, in the presence of a reducing agent (GSH), the delivery systems based on the K(HC)EF-1 and K(HC)F-1 polypeptides demonstrated rapid destruction of the complexes due to the reduction of disulfide bonds. The loss of nanoparticle stability led to aggregation within the first hours. At the same time, a pDNA delivery system based on K(HC)EF was stable in PBS and DMEM + FCS for 4 and 5 days (Figure 7a), respectively, while the K(HC)F-based delivery system was stable within the experiment (7 days) in both media (Figure 7b).

Furthermore, the stability of pDNA-loaded cross-linked nanoparticles was also examined using heparin displacement test in the absence and presence of GSH by agarose gel electrophoresis. Heparin is a strong natural polyanion that can compete with nucleic acid for binding with polycations. In our case, a different amount of heparin solution was added to a dispersion of the pDNA-loaded cross-linked nanoparticles to reach the ratios for the –/+ charges in the range of 0.5 to 20.

**Figure 8:**
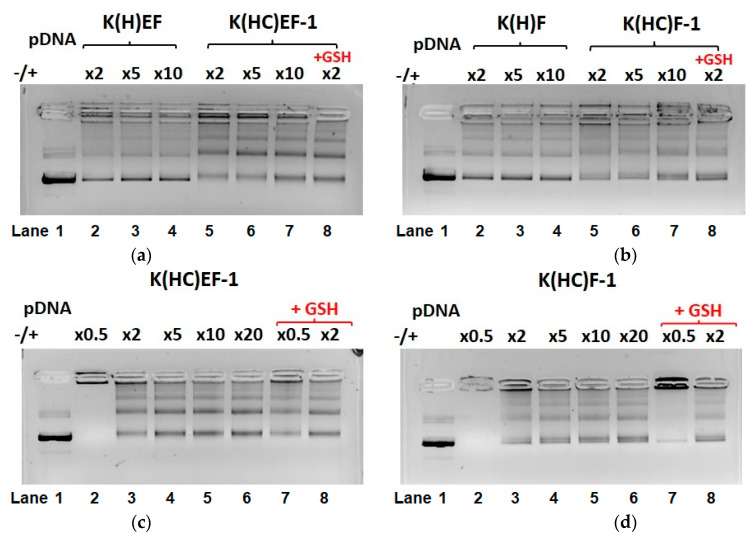
Heparin displacement test: (**a**) comparison of stability of pDNA complexes with precursor K(H)EF polypeptide and the S-S cross-linked K(HC)EF polypeptide nanoparticles at different excesses of heparin; (**b**) comparison of stability of pDNA complexes with precursor K(H)F polypeptide and the S-S cross-linked K(HC)F polypeptide nanoparticles at different excesses of heparin; (**c**) effect of different concentrations of heparin and glutathione on the stability of pDNA complexes with the S-S cross-linked K(HC)EF polypeptide nanoparticles; (**d**) effect of different concentrations of heparin and glutathione on the stability of pDNA complexes with the S-S cross-linked K(HC)F polypeptide nanoparticles.

First, the effect of cross-linking on the stability of displacement of pDNA by heparin was investigated. For this purpose, cross-linked pDNA delivery systems were compared with those obtained using cysteine-free parent polypeptides (K(H)EF and K(H)F) (Figure 8a,b). The displacement of pDNA by heparin was observed at all charge ratios for both cross-linked and non-cross-linked nanoparticles. However, while it was effective and did not depend on the heparin concentration for non-cross-linked nanoparticles (Figure 8a,b, lanes 2–4), for cross-linked systems, the pDNA displacement was less effective and evidently dependent on the polyanion excess (Figure 8a,b, lanes 5–7). Changing the −/+ charge ratios from 0.5 to 20 revealed that at a ratio of 0.5, no displacement of pDNA occurred for both kinds of cross-linked polypeptide nanoparticles (whether containing glutamic acid or not) (Figure 8c,d, lanes 2). At the same time, an increase in the −/+ charge ratio from 2 to 20 was accompanied with an increase in the amount of displaced pDNA (Figure 8c,d, lanes 3–6).

Second, the effect of glutathione addition simultaneously with heparin was studied for cross-linked pDNA delivery systems. For both K(H)EF- and K(H)F-based delivery systems, the addition of GSH stimulated the partial displacement of pDNA by heparin even at the −/+ charge ratio of 0.5 (Figure 8c,d, lane 7). Since no displacement of pDNA was observed under the same conditions without the reducing agent; this displacement is a result of disulfide bond reduction and loosening of the nanoparticle, facilitating displacement by heparin. However, a comparison of lanes 7 in Figure 8c,d allowed for the conclusion that the release of pDNA from nanoparticles self-assembled from polypeptides containing glutamic acid, namely, K(HC)EF, is more pronounced than from nanoparticles based on K(HC)F polypeptide. This effect is explained by the facilitation of nucleic acid release in the presence of negatively charged amino acid. Recently, a similar trend was observed for the release of siRNA from non-cross-linked nanoparticles containing and not containing a negatively charged amino acid [29]. Increasing the −/+ charge ratio from 0.5 to 2 at the same GSH concentration additionally promoted the release of pDNA (Figure 8c,d, lanes 8).

Thus, the intraparticle cross-linking of polypeptides by disulfide bonds allowed for the formation of nanoparticles to be more stable in the presence of competitive polyanions. In addition, the presence of redox-sensitive cross-links reduced the degree of pDNA release in the presence of competing polyanions but did not prevent release after the destruction of the stabilizing cross-links.

### 2.6. Cytotoxicity and pDNA Delivery into HEK 293 Cells

**Figure 9:**
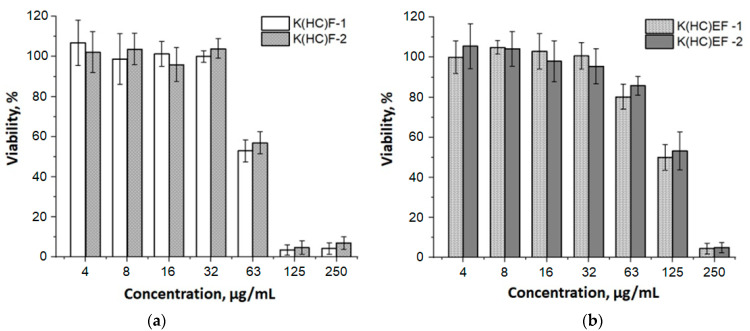
Viability of HEK 293 cells after incubation with samples K(HC)F-1 and K(HC)F-2 (**a**), and K(HC)EF-1 and K(HC)EF-2 (**b**) over 48 h. The data are shown as the mean ± SD (*n* = 4).

Cytotoxicity evaluation of the polymeric carrier is a necessary step before its further testing in vitro and in vivo. In this study, the cellular relative viability was evaluated by the MTT assay using the human embryonic kidney (HEK 293) cell line. The cells were exposed to K(HC)EF-1, K(HC)EF-2, K(HC)F-1 and K(HC)F-2 polypeptide nanoparticles for 48 h; untreated cells were used as references. It was established that all samples did not provide cytotoxicity within the range of concentrations between 4 and 32 µg/mL (Figure 9). Moreover, both samples containing glutamic acid, namely K(HC)EF-1 and K(HC)EF-2, were not toxic at a concentration of 63 μg/mL. Lower toxicity may be explained by the decrease in lysine content as well as compensation in the part of lysine with glutamic acid in K(HC)EF nanoparticles in comparison to K(HC)F-1 and K(HC)F-2.

**Figure 10:**
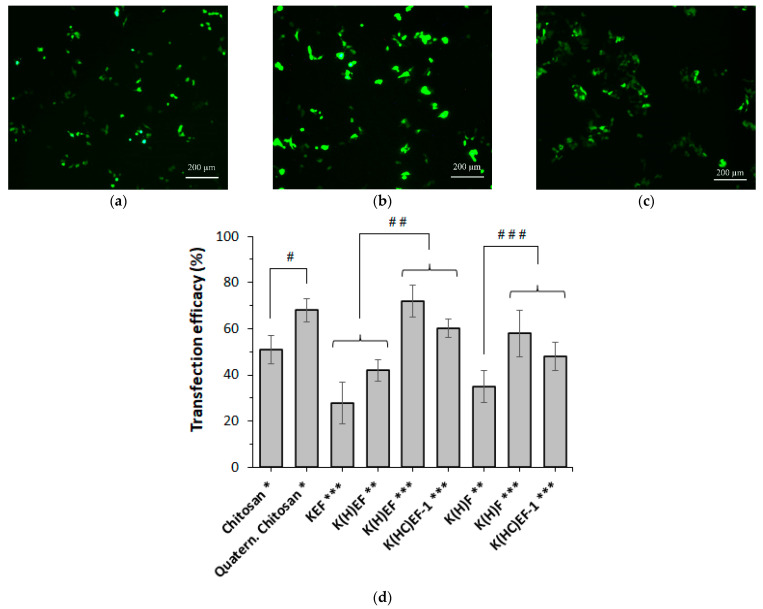
Transfection of pEGFP-N3 to HEK 293 cells with the use of various polymer delivery systems: (**a**,**b**) Fluorescence microscopy after 5 days of cell incubation with polymer/pDNA complexes (GFP channel, ×10). Samples: chitosan (**a**), K(H)EF (**b**) and K(HC)F-1 (**c**); (**d**) comparative transfection efficacy for the tested polymer/pEGFP-N3 systems. pDNA content: *—500 ng (polymer/pDNA mass ratio was 100:1), **—100 ng (polymer/pDNA mass ratio was 50:1), ***—200 ng (polymer/pDNA mass ratio was 100:1). Statistics: #—*p* ˂ 0.05, ##—*p* ˂ 0.005, ###—*p* ˂ 0.008.

The transfection efficacy of the polymer carriers was evaluated using the HEK 293 cell line, which initially did not produce green fluorescent protein (GFP). Plasmid pEGFP-N3 (4729 base pairs), which encodes the GFP protein, was selected as a genetic construct. Transfected cells were evaluated using fluorescence microscopy (Figure 10), and cells transfected with pEGFP without polymer carriers and cells transfected with empty polymer nanoparticles were used as controls. The amount of pDNA per well was 100 or 200 ng, according to the standard protocol. Microscopy of cells was performed after 5 days of cell incubation with pDNA-loaded nanoparticles. Poly(*L*-lysine), chitosan and quaternized chitosan (*N*-[4-(*N*′,*N*′,*N*′-trimethylammonium)benzyl]chitosan) [36] were used as control polycations for pDNA delivery.

As expected, no transfection was observed when free pEGFP and empty polymer nanoparticles were used in the experiment. In addition, delivery of pDNA in a complex with homopolymer poly(*L*-lysine) at a concentration higher than 15 µg/mL was unsuccessful due to high toxicity of the polymer and death of more than 70% of cells. The application of polyelectrolyte complexes chitosan/pDNA and quaternized chitosan/pDNA resulted in successful cell transfection equal to 50 and 70% (*p* ˂ 0.05), respectively. The experiment was carried out using 500 ng pDNA per well and polymer/pDNA ratio equal to 100:1. Such loading was previously found to be optimal by Badazhkova et al. [36]. The authors showed that a large excess of cationic polymer was required to reach a sufficient transfection level. However, chitosan and its derivative were non-toxic even at a high concentration.

Recently, the histidine-containing polypeptide nanoparticles had proven their effectiveness as siRNA carriers [29]. In this study, cysteine-free polypeptide systems, as well as cysteine-containing carriers stabilized by disulfide bonds, were investigated for the intracellular delivery of pDNA. Parent (KEF and KF), and their pH-responsive (K(H)EF and K(H)F) and both pH- and redox-responsive (K(HC)EF and K(HC)F) derivatives were compared. Since the composition, physicochemical properties and cytotoxicity of the samples 1 and 2 in the series of K(HC)EF and K(HC)F were very close, the transfection efficacy was evaluated for only K(HC)EF-1 and K(HC)F-1 samples. The lowest transfection efficacy (~30%) was found for parent KEF polypeptide (Figure 10d), while the delivery of pDNA by the KF system was not possible to evaluate due to high cytotoxicity of the polypeptide (IC_50_ = 16 µg/mL).

An increase in the amount of pDNA from 100 to 200 ng of pDNA per well for the His-containing nanoparticles promoted enhanced transfection (polypeptide:pDNA mass ratio of 50:1 or 100:1). A comparison of His-containing polypeptides with and without glutamic acid in the structure allowed for the conclusion of a slightly higher transfection of the latter (by approximately 14%) (Figure 10d). This result may be related to the features of pDNA localization (Figure 6, Section 2.4) and its further, more efficient, release from the Glu-containing polypeptides. In turn, the introduction of Cys and intraparticle S-S cross-linking resulted in a slight decrease in transfection efficacy (approximately by 10–12%). This tendency may be the result of less flexibility of the polymeric structure affecting cell entrance and the rate of nucleic acid release from the polypeptide. Despite this, the combination of a fairly efficient transfection and extracellular stability of the K(HC)EF and K(HC)F cross-linked systems makes them promising for further, more in-depth, research. In comparison to chitosan, the developed polypeptide delivery systems containing 200 ng pDNA demonstrated the transfection efficacy similar to chitosan and its quaternized derivative loaded with 500 ng pDNA (Figure 10d).

Thus, it was demonstrated that pH- and redox-responsive polypeptides had good stability in different media, were able to penetrate inside a cell, and efficiently transferred a gene. Moreover, they were less toxic than well-known poly(L-lysine) and transfected the cells efficiently at lower amounts of pDNA than chitosan and its quaternized derivative.

## 3. Materials and Methods

### 3.1. Materials

ε-Z-*L*-lysine, *L*-glutamic acid γ-benzyl ester, *N-α*-Fmoc-*N*′-trityl-*L*-histidine, *L*-phenylalanine, triphosgene, α-pinene, *tert*-butylamine, trifluoroacetic acid (TFA), trifluoromethanesulfonic acid (TFMSA), 5,5’-dithiobis-(2-nitrobenzoic acid), *N,N*’-Diisopropylcarbodiimide (DIC, 99%), *N*-hydroxysuccinimide (NHS, 98%), dimethyl sulfoxide-d6 (DMSO-d6, 99.8%), *N*_α_-Fmoc-*S*-acetamidomethyl-L-cysteine pentafluorophenyl ester (Fmoc-Cys(Acm)-OPfp) and other reagents for synthesis were obtained from Sigma–Aldrich (Darmstadt, Germany) and used without additional purification. All organic solvents, i.e., *N,N*-dimethylformamide (DMF), diethyl ether, 1,4-dioxane, dimethyl sulfoxide (DMSO), ethyl acetate, petroleum ether and some others were ordered from Vecton Ltd. (St. Petersburg, Russia), and purified before use according to standard protocols. For purification of synthesized polymers, Spectra/Pore^®^ dialysis bags (MWCO:1000, Rancho Dominguez, CA, USA) were used. Amicon Ultra filter tubes with 3000 MWCO (0.5 mL) were purchased from Merck (Darmstadt, Germany).

Plasmid DNA bearing green fluorescent protein gene (pEGFP-N3) was the product of Clontech Takara Bio (supplied by Axioma Bio Ltd., Moscow, Russia). pEGFP-N3 was used in the biological experiments. The detailed information on the used plasmid can be found in the AddGene Vector Database [37]. pEGFP-C2, kindly donated by colleagues from the University of Eastern Finland (Kuopio, Finland), was used for the binding study with polymer carriers. Chitosan and quaternized chitosan derivative were kindly provided by Dr. Yu. Skorik from IMC RAS. Human embryonic kidney cell line (HEK 293) was obtained from Cell line collection of Institute of Cytology of Russian Academy of Sciences (St. Petersburg, Russia). All other materials are described further upon their appearance in the text.

### 3.2. Methods

#### 3.2.1. Synthesis and Characterization of Polypeptides

Polypeptide synthesis via ring-opening polymerization (ROP) of α-amino acid N-carboxyanhydrides was carried out according to the previously published procedure [29]. Briefly, NCA monomers of Lys(Z), Glu(OBzl) and Phe were prepared via the reaction of α-amino acids with triphosgene under inert atmosphere in anhydrous dioxane with addition of α-pinene. Synthesized NCAs were purified by recrystallization from anhydrous ethyl acetate/petroleum ether.

^1^H NMR spectroscopy of monomers and polymers was performed using a Bruker AC-400 NMR spectrometer (400 MHz) (Karlsruhe, Germany) at 25 °C in DMSO-d_6_. ^1^H NMR of monomers (CDCl_3_, 25 °C), δ (ppm): Lys(Z) NCA: 7.43–7.28 (m, 5H), 6.97 (s, 1H), 5.12 (s, 2H), 4.97 (s, 1H), 4.32–4.23 (t, *J* = 5.2, 1H) (s, 1H), 3.29–3.14 (m, 2H), 2.03–1.90 (m, 1H), 1.90–1.75 (m, 1H), 1.73–1.28 (m, 4H); Glu(OBzl) NCA: 2.05–2.39 (m, 2H), 2.63 (t, 2H), 4.39 (t, 1H), 5.17 (s, 2H), 6.40 (br. s., 1H), 7.39 (m, 5H); Phe NCA: 2.94–3.35 (m, 2H), 4.55 (m, 1H), 6.12 (s, 1H), 7.19–7.41 (m, 5H); Ile NCA: 0.836 (t, 3H), 0.871 (d, 3H), 1.236 (dq, 2H), 1.941 (qtd, 1H), 4.28 (d, 1H). The yields of Lys(Z) NCA, Glu(OBzl) NCA and Phe NCA were 76, 80 and 63%, respectively.

The monomers obtained were used to synthesize two polypeptides, namely P(Lys(Z)-*co*-Glu(OBzl)-*co*-Phe) and P(Lys(Z)-*co*-Phe). The used ratios of NCAs were Lys(Z)/Glu(OBzl)/Phe = 70/15/15 and Lys(Z)/Phe = 75/25. The NCAs/initiator (*n*-hexylamine) ratio was 100. The polymerization was carried out using 4 wt% solution of NCAs in 1,4-dioxane for 48 h at 25 °C. ^1^H NMR (DMSO-d6, 25 °C), δ (ppm): NH_2_-C_6_H_11_: 0.84 (CH_3_); Lys: 2.97 (NH-CH_2_), 4.1–4.34 (CH), Z-group: 5.0 (O-CH_2_-C_6_H_5_) 7.08–7.42 (O-CH_2_-C_6_H_5_); Phe: 4.6 (CH-CH_2_-C_6_H_5_) и 6.9–7.2 (C_6_H_5_); Glu: 4.26 (CH); OBzl-group: 5.03 (O-CH_2_-C_6_H_5_) и 7.33 (O-CH_2_-C_6_H_5_); Ile 0.85 (CH_3_), 4.28 (CH). The yields of the P(Lys(Z)-*co*-Glu(OBzl)-*co*-Phe) and P(Lys(Z)-*co*-Phe) were 65 and 76%, respectively.

Deprotection of copolymers was achieved by incubation in 9% TFMSA in TFA 30 min when cooled in an ice bath and 4 h at room temperature. The products were precipitated with diethyl ether, and washed by dialysis against water for 36 h. The composition of obtained polypeptides was determined using the quantitative HPLC amino acid analysis performed after total hydrolysis of polypeptides up to free amino acids according to the previously published procedure [28]. Weight average molecular-weight (*M_w_*) and macromolecule hydrodynamic diameter (*R_h-D_*) were determined by the static and dynamic light scattering as described earlier [29].

Purified deblocked copolymers were modified with L-histidine using Fmoc(His)Trt-OH with previously activated carboxylic group. Activation was carried out using DIC and NHS in DMF [29]. Fmoc(His)Trt-ONHS was taken as 60 mol% of the molar amount of lysine in the copolymers. The modification was carried out in DMF for 2 h at 22 °C. The copolymers were purified by dialysis against DMF/H_2_O mixture (50/50, *v*/*v*) and then H_2_O for 36 h. In the ^1^H NMR (DMSO-d_6_) spectra of the modified copolymers, a significant increase in the signal intensity in the 7.1–7.9 ppm was observed due to the signals from protons of the imidazole ring as well as due to the bands from protons of benzene rings of protective groups Fmoc and Trt (C_6_H_5_).

To prepare redox-sensitive copolymers, the polypeptides were modified with L-cysteine. For this purpose, a cysteine derivative with an activated carboxylic group and blocked thio- and amino groups, namely, *Nα*-Fmoc-S-(Acm)-*L*-cystein pentafluorophenyl ester (Fmoc-Cys(Acm)-OPfp) was used. Solutions of copolymers P(Lys-*co*-Lys(Fmoc-His(Trt))-*co*-Phe) and P(Lys-*co*-Lys(Fmoc-His(Trt))-*co*-Glu-*co*-Phe) in DMF (0.01 mg/mL) were prepared. After that, the solution of Fmoc-Cys(Acm)-OPfp in DMF was added dropwise to the flasks and left stirring on a magnetic stirrer overnight at room temperature. The amount of added cysteine derivative was calculated relative to ε-amino groups of lysine and equal to 7 or 12 mol%. The resulting product was purified from low molecular weight compounds by dialysis against DMF/H_2_O mixture (50/50, *v*/*v*) and then H_2_O. The modification of copolymers with Cys derivative was testified by the appearance of a new signal at 1.9 ppm of Acm-protective group (CH_3_CO-) in ^1^H NMR (DMSO-d6) spectra.

The protective groups of histidine and cysteine (Fmoc, Trt, and Acm) were removed. Firstly, Fmoc-protective groups were cleaved by addition of 20% of *tert*-butylamine solution in DMF for 16 h at 22 °C. The resulting products were precipitated with a five-fold excess of cooled diethyl ether. After that, each copolymer was placed separately in an ice-bath and dissolved in TFA to remove the Trt-protective group. The reaction ran for 1.5 h. The deprotected products were precipitated with five-fold excess of cooled diethyl ether and then centrifuged. The resulting precipitates were dissolved in 5 mL of 30 vol% acetic acid to cleave the Acm-protective group; then, the solution was purged with argon, and a two-fold excess of mercury acetate relative to the amount of cysteine was added. The reaction mixtures were left stirring on a magnetic stirrer for 1 h at room temperature. Then, a given volume of β-mercaptoethanol was added, and the solutions were stirred for 2 h more. The resulting products were purified by dialysis for 24 h against distilled water and then lyophilized. The yields of the modified polypeptides were in the range of 60–76%. The amount of thiol groups was determined by the Ellman’s method with the use of 5,5’-dithiobis-(2-nitrobenzoic acid) (DTNB) as described elsewhere [38].

The dry polypeptide samples were stored at 4 °C.

#### 3.2.2. Preparation and Characterization of Nanoparticles

The obtained polypeptides are amphiphilic and have a tendency to self-assemble in aqueous media. The self-assembly of polypeptides was already achieved during dialysis (a gradient solvent inversion) from organic solvent to water during a previous step (Section 3.2.1) followed with freeze-drying. To prepare the dispersion of nanoparticles, the weighted sample of nanoparticles was dispersed in water or buffer solution under short-term ultrasonic exposure (30 s).

To prepare cross-linked nanoparticles, the dispersion of polymer nanoparticles particles in water or buffer solution was diluted to a desired concentration. Secondly, 100 μL of 10 mM GSH solution was added to 900 μL dispersion of nanoparticles (final concentration of nanoparticles was 1 mg/mL) to reduce possible S-S bonds formed due to possible thiol oxidation during nanoparticle storage. After 30 min of incubation, the suspension was ultrafiltrated and washed several times using Amicon Ultra filter tubes with 3000 MWCO in order to remove glutathione. Then, the suspension was ultrasonicated once more. Oxidation of the thiol groups was performed using gaseous oxygen. The gas was bubbled through a given volume of solution for 20 min and then incubated for 2 h at room temperature. After that, the cross-linked nanoparticles were characterized.

The hydrodynamic diameter and ζ-potential of polypeptide nanoparticles were measured by dynamic light scattering (DLS) and electrophoretic light scattering (ELS) methods. ZetasizerNano-ZS (Malvern, UK) equipped with a He–Ne laser at 633 nm at a scattering angle of 173° and 25 °C was used for analysis. All measurements were performed at least 3 times. For measurements, water or aqueous buffer solutions with a concentration of nanoparticles equal to 0.1 mg/mL were utilized.

#### 3.2.3. Preparation of pDNA-Loaded Cross-Linked Nanoparticles

Negatively charged pDNA was easily bound to positively charged polypeptides by means of electrostatic interactions between phosphate groups and free amino groups of lysine or histidine. Firstly, the dispersion of polymer particles was prepared as described in Section 3.2.2. Then, the suspension was ultrasonicated once more. Finally, the solution of pDNA was added to the suspension under stirring (Vortex, Thermo Fischer Scientific, Vantaa, Finland). The mixture was incubated for 30 min at room temperature for stabilization of complexes.

Oxidation of the thiol groups was performed using gaseous oxygen. The gas was bubbled through the dispersion containing pDNA-loaded nanoparticles during 20 min, followed by complex incubation for 2 h at room temperature (22 °C). After that, the cross-linked complexes were investigated via DLS and ELS or used in further cell experiments.

#### 3.2.4. Investigation of Encapsulation Efficacy and Complexes Stability

The encapsulation efficacy of pDNA-loaded nanoparticles was assessed by dynamic light scattering and agarose gel electrophoresis. For this purpose, a number of samples were prepared, including complexes with pEGFP-C2 without cross-linking and complexes stabilized by disulfide bonds. The polypeptide:pDNA mass ratios were varied from 50:1 or 100:1. For the gel electrophoresis analysis, a 10 µL sample solution was mixed with 4 µL of dye (xylene blue and bromophenol blue, Thermo Fisher Scientific, Waltham, MA, USA) and loaded into a 1% agarose gel (containing SYBR Safe dye, Thermo Fisher Scientific, Waltham, MA, USA) in EDTA trisborate buffer solution, pH 8.0. Electrophoresis of the samples was performed at an operating voltage of 100 V for 1.5 h. Then, the gel was analyzed using the Gel Doc EZ gel documentation system (BioRad, Hercules, CA, USA). The images of the original gels presented in Figure 6 are provided in Figure S1 (Supplementary Materials).

The heparin displacement test was carried out to investigate the binding between polypeptides and pDNA in the presence of competing polyanion. Polymer–pDNA complexes were prepared at different mass ratios; then, a certain volume of heparin solution was added to obtain a 0.5–20-fold excess of −/+ charge. In addition, the sensitivity of the pDNA-loaded cross-linked nanoparticles to GSH was investigated at the GSH concentration of 0.5 and 2 mM. The images of the original gels presented in Figure 8 are provided in Figure S2 (Supplementary Materials).

#### 3.2.5. Cytotoxicity

HEK 293 cell line was cultivated in 75 cm^2^ culture vials under mycoplasma-free conditions in DMEM-F12, containing 10% (*v*/*v*) fetal bovine serum and 1% (*v*/*v*) penicillin/streptomycin. The cytotoxicity of the polymer particles toward HEK 293 cell line was evaluated by a 3-(4,5-dimethylthiazol-2-yl)-2,5-diphenyltetrazolium bromide (MTT) reduction assay. For this, HEK 293 cell line was seeded into a 96-well plate in the amount of 10^4^ cells in 200 mL of culture medium per well and incubated at 37 °C overnight. Then, a suspension of polymer particles was added to each well so that their final concentration varied from 4 to 250 μg/mL. Further, the cells were incubated with the particles for 48 h; after that, the viability of the cells was evaluated using MTT analysis. For this, a culture medium was aspirated from the wells, and 100 µL/well of MTT solution (5 mg/mL in DMEM-F12) was added; the cells were incubated for 2 h. Then, the solution was carefully removed, and 50 µL DMSO was added to each well to lyse the cells and dissolve the crystals of farmazan salt. Absorption of solution (A) was measured at 570 nm using a Fluoroskan Ascent plate reader (Thermo Fisher Scientific, Waltham, MA, USA). All experiments were carried out at least 3 times. Intact cells were not added and were used as positive control (A_control_), PBS was used as solvent control (A_blank_). Relative cell viability (%) was calculated the following way:

(1) $$\mathrm{Cell}\;\mathrm{viability}=\frac{A-A_{\mathrm{blank}}}{A_{\mathrm{control}}-A_{\mathrm{blank}}}\times 100\%$$

Cell viability less than 75% indicated sample toxicity.

#### 3.2.6. Induction of GFP Protein Expression

HEK 293 cells were seeded in a 96-well plate at a concentration of 10^3^ cells per well in 100 µL of DMEM-F12 medium with serum and antibiotics. After 15 h, the cell medium was changed with 200 µL of DMEM-F12 serum-free medium. Then, 20 µL of dispersion containing 100–200 ng of pEGFP-N3 (4729 base pairs) complexed with a polymer (polymer:pDNA mass ratios = 50:1 or 100:1) (were added to each well. For chitosan and its derivative 500 ng of pDNA was used for transfection. After 4 h of incubation at 37 °C, the medium was changed back to the serum-containing medium. The experiment took 5 days. The transfection efficiency was analyzed by fluorescence microscopy, estimating the number of cells containing GFP protein relative to the total number of cells that could be seen in the microscope.

#### 3.2.7. Statistical Analysis

The data are presented as mean value ± SDV (*n* = 4 for biological experiments; *n* = 3 for physicochemical experiments). Statistically significant differences were analyzed by two-way ANOVA, using Instat (GraphPad Software Inc., San Diego, CA, USA). *p* < 0.05 was considered as statistically significant.

## 4. Conclusions

In this study, the novel pH- and redox-sensitive gene delivery systems based on biodegradable carriers were designed, synthesized and investigated. Nanoparticles based on histidine and cysteine-containing amphiphilic lysine-enriched polypeptides were able to self-assembly into nanoparticles. The hydrodynamic diameter of S-S-cross-linked nanoparticles were in the range of 55–100 nm. The developed nanoparticles were stable in buffer and protein-containing media but were easily destroyed in the presence of intracell reducing agent (glutathione). Polypeptides were able to bind pDNA effectively at polypeptide/pDNA ratios of 10 and higher. The formed delivery systems were stable and released cargo in the presence of competitive polyanion (heparin) and glutathione. The glutamic acid-free nanoparticles were non-cytotoxic up to 32 μg/mL and in nanoparticles containing glutamic acid up to 63 μg/mL. The different delivery systems provided efficient transfection of pEGFP into HEK 293, resulting in 30–70% expression of GFP depending on polypeptide and pDNA content. Thus, the developed pH- and redox-responsive polypeptide nanoparticles can be considered as stable promising gene delivery systems that protect DNA from premature release and provide pH-sensitive escape from endosomes with subsequent release as a result of disulfide bond reduction.

## Figures and Tables

**Table 1:** Compositions of the synthesized stimuli-responsive polypeptides.

Sample	Composition of Polypeptide Backbone (mol %)			Composition of Grafted Amino Acids (mol% from Lys Content)	
	Lys	Glu	Phe	His *	Cys *
P(Lys-co-Lys(His)-co-Lys(Cys)-co-Glu-co-Phe) **					
K(HC)EF-1	70	15	15	50	5
K(HC)EF-2	70	15	15	50	8
P(Lys-co-Lys(His)-co-Lys(Cys)-co-Phe) **					
K(HC)F-1	77	-	23	48	5
K(HC)F-2	77	-	23	48	8

* The amount of grafted histidine and cysteine was calculated relative to the amount of lysine taken as 100%; ** Samples numbered 1 and 2 differ in the initial amount of Cys taken for the reaction. The initial amount of Cys was 7 mol% for sample #1 and 12 mol% for sample #2.

**Table 2:** Physicochemical parameters of the nanoparticles in water.

Sample	*D_H_* (nm)	PDI	ζ-Potential (mV)
*P(Lys-co-Lys(His)-co-Lys(Cys)-co-Glu-co-Phe)*			
K(HC)EF-1	78 ± 34	0.41	+61
K(HC)EF-2	55 ± 22	0.43	+63
*P(Lys-co-Lys(His)-co-Lys(Cys)-co-Phe)*			
K(HC)F-1	92 ± 33	0.37	+58
K(HC)F-2	100 ± 38	0.40	+65

## Data Availability

Data are available within the article.

## Supplementary Materials

The following supporting information can be downloaded at: https://www.mdpi.com/article/10.3390/molecules27238495/s1, Figure S1: The original gel-electrophoresis images to the marked ones shown in Figure 6 of the main text. The description is provided in Figure 6 and its legend, as well as in the text of Section 2.4; Figure S2: The original gel-electrophoresis images to the marked ones shown in Figure 8 of the main text: top and bottom images (a) correspond to (a) and (b) images of Figure 8, respectively; top and bottom images (b) correspond to (c) and (d) images of Figure 8, respectively. The description is provided in Figure 8 and its legend, as well as in the text of Section 2.5.

## Sample Availability

Samples of the polymers are available from the authors on request.
