# Supplementary material for: Stimuli-Responsive Polypeptide Nanoparticles for Enhanced DNA Delivery
Source: Molecules. 2022 Dec 2;27(23):8495. doi: 10.3390/molecules27238495 (PMC9736633; doi:10.3390/molecules27238495)
Supplement: Supplementary file 1 [file molecules-27-08495-s001.zip › molecules-2023334-supplementary.pdf]

molecules

Supplementary Materials

Stimuli-Responsive Polypeptide Nanoparticles for Enhanced DNA delivery

Olga Korovkina [1], Dmitry Polyakov [2], Viktor Korzhikov-Vlakh [1,3] and Evgenia Korzhikova-Vlakh [3]*

[1] Saint-Petersburg State University, Institute of Chemistry, Universitetsky pr. 26, 198504 St. Petersburg, Russia
[2] Institute of Experimental Medicine, Acad. Pavlov Street 12, 197376 St. Petersburg, Russia
[3] Institute of Macromolecular Compounds, Russian Academy of Sciences, Bolshoy pr. 31, 199004, St. Petersburg, Russia
* Correspondence: vlakh@hq.macro.ru (E.K.-V.)

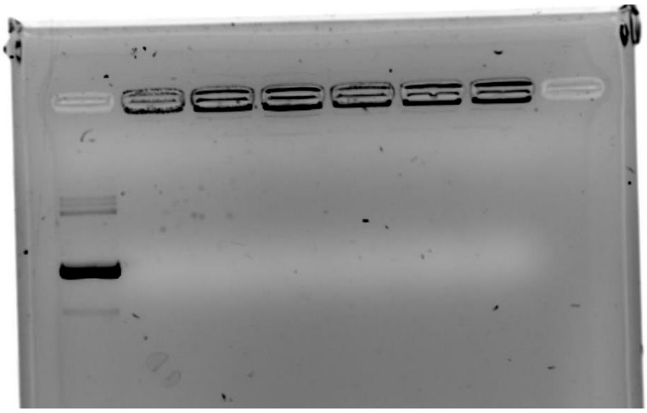

(a)

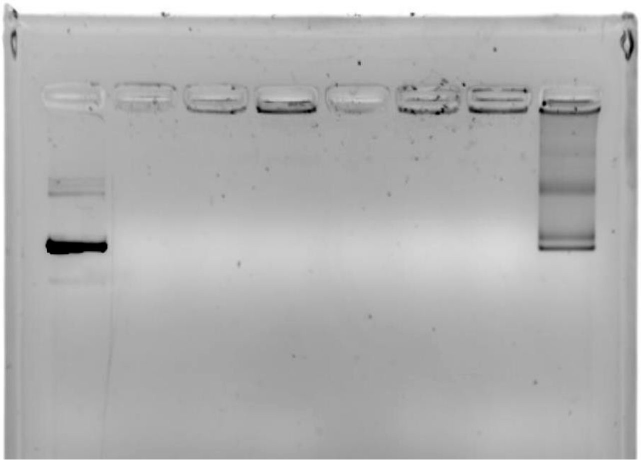

(b)

Figure S1. The initial gel-electrophoresis images to the marked ones shown in Figure 6 of the main text: (a) and (b) correspond to (a) and (b) of Figure 6. The description is provided in the Figure 6 and its legend, as well as in the text of section 2.4.

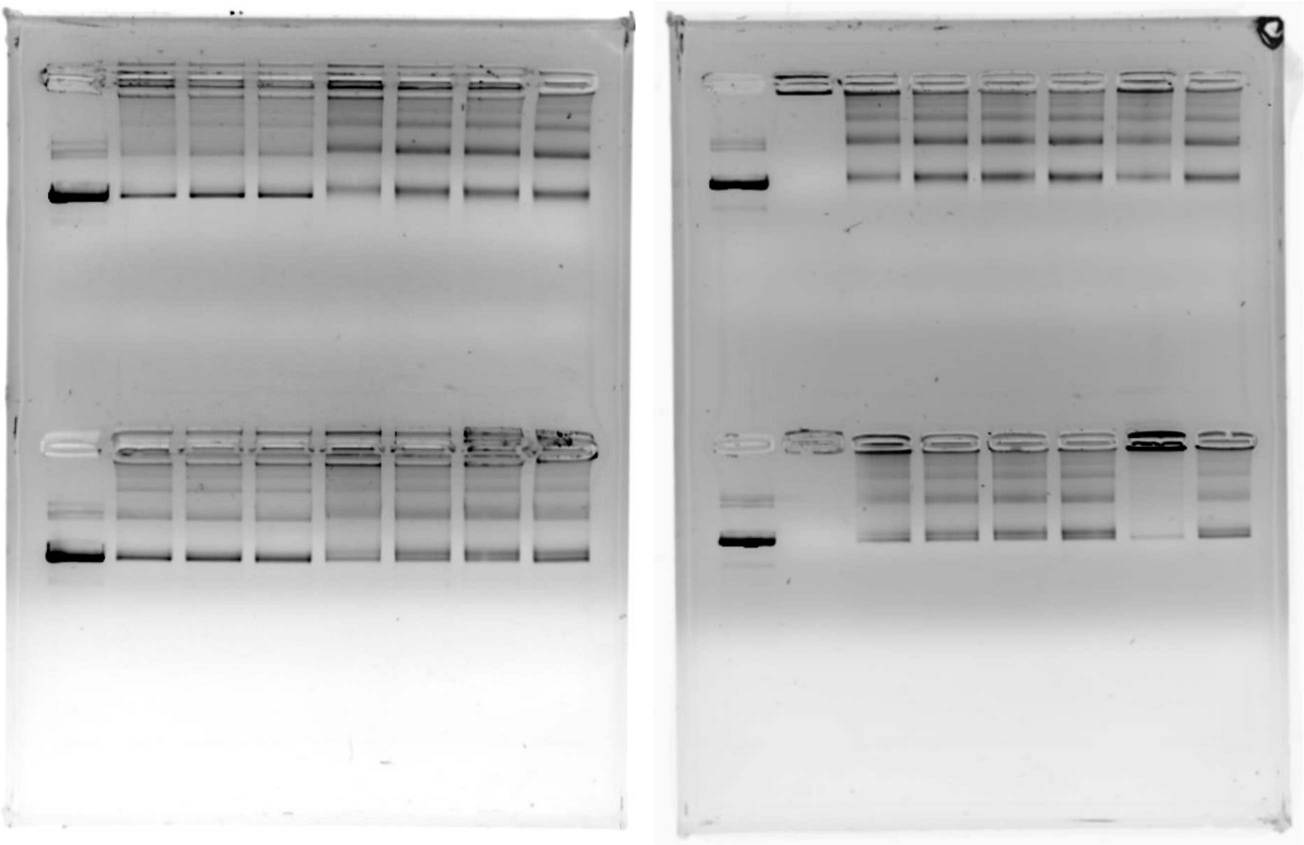

(a) (b)

Figure S2. The initial gel-electrophoresis images to the marked ones shown in Figure 8 of the main text: top and bottom images **(a)** correspond to (a) and (b) images of Figure 8, respectively; top and bottom images **(b)** correspond to (c) and (d) images of Figure 8, respectively. The description is provided in the Figure 8 and its legend, as well as in the text of section 2.5.
